# Pathophysiological Processes Underlying the High Prevalence of Deep Vein Thrombosis in Critically Ill COVID-19 Patients

**DOI:** 10.3389/fphys.2020.608788

**Published:** 2021-01-08

**Authors:** Sebastian Voicu, Chahinez Ketfi, Alain Stépanian, Benjamin G. Chousterman, Nassim Mohamedi, Virginie Siguret, Alexandre Mebazaa, Bruno Mégarbane, Philippe Bonnin

**Affiliations:** ^1^Department of Medical and Toxicological Critical Care, Hôpital Lariboisière, APHP, Faculté de Santé, Université de Paris, Paris, France; ^2^INSERM UMRS 1144, Université de Paris, Paris, France; ^3^Department of Clinical Physiology, Hôpital Lariboisière, APHP, Faculté de Santé, Université de Paris, Paris, France; ^4^Laboratory of Biological Hematology, Hôpital Lariboisière, APHP, Faculté de Santé, Université de Paris, Paris, France; ^5^EA3518, Université de Paris, Paris, France; ^6^Department of Anesthesia and Critical Care, Faculté de Santé, Hôpital Lariboisière, APHP, FHU PROMICE, Paris, France; ^7^Université de Paris, Paris, France; ^8^INSERM UMR-S 942 – MASCOT, Université de Paris, Paris, France; ^9^INSERM UMR-S 1140, Université de Paris, Paris, France; ^10^INSERM U1148, LVTS, Université de Paris, Paris, France

**Keywords:** COVID-19, D-dimer, hemostasis disorder, deep vein thrombosis, venous stasis

## Abstract

Coronavirus disease 2019 (COVID-19) predisposes to deep vein thrombosis (DVT) and pulmonary embolism (PE) particularly in mechanically ventilated adults with severe pneumonia. The extremely high prevalence of DVT in the COVID-19 patients hospitalized in the intensive care unit (ICU) has been established between 25 and 84% based on studies including systematic duplex ultrasound of the lower limbs when prophylactic anticoagulation was systematically administrated. DVT prevalence has been shown to be markedly higher than in mechanically ventilated influenza patients (6–8%). Unusually high inflammatory and prothrombotic phenotype represents a striking feature of COVID-19 patients, as reflected by markedly elevated reactive protein C, fibrinogen, interleukin 6, von Willebrand factor, and factor VIII. Moreover, in critically ill patients, venous stasis has been associated with the prothrombotic phenotype attributed to COVID-19, which increases the risk of thrombosis. Venous stasis results among others from immobilization under muscular paralysis, mechanical ventilation with high positive end-expiratory pressure, and pulmonary microvascular network injuries or occlusions. Venous return to the heart is subsequently decreased with increase in central and peripheral venous pressures, marked proximal and distal veins dilation, and drops in venous blood flow velocities, leading to a spontaneous contrast “sludge pattern” in veins considered as prothrombotic. Together with endothelial lesions and hypercoagulability status, venous stasis completes the Virchow triad and considerably increases the prevalence of DVT and PE in critically ill COVID-19 patients, therefore raising questions regarding the optimal doses for thromboprophylaxis during ICU stay.

## Introduction

Coronavirus disease 2019 (COVID-19) patients present with coagulation disorders and marked predisposition to thrombosis (Goshua et al., 2020; Helms et al., 2020; Iba et al., 2020). One major consequence is the high prevalence of deep vein thrombosis (DVT) demonstrated by studies performing duplex ultrasound examination of the lower limbs in patients hospitalized with severe pneumonia (Zhang et al., 2020; Fraissé et al., 2020; Llitjos et al., 2020; Nahum et al., 2020; Ren et al., 2020; Voicu et al., 2020a). The high prevalence of DVT exposes patients to a high risk of pulmonary embolism (PE) (Contou et al., 2020; Poissy et al., 2020), especially when DVT is proximal, at the popliteal level, or above (Konstantinides et al., 2020). DVT in COVID-19 patients may be responsible for approximately 10% of deaths in COVID-19 patients as shown by systematic autopsy findings (Edler et al., 2020). Therefore, understanding the underlying mechanisms of the thrombotic process during the COVID-19 time course is of utmost importance because of the implications in the clinical, biological, and imaging monitoring of the patients, as well as in the anticoagulant prophylaxis and treatment.

### High Prevalence of DVT in COVID-19 Patients—Clinical Data

Initial studies reported unusually high rates of thrombotic events including DVT and PE, i.e., 16.7% (Helms et al., 2020), 20.6% (Poissy et al., 2020); DVT, 2% (Helms et al., 2020), 14.8% (Tavazzi et al., 2020); and DVT and/or PE in 27% of the patients (Klok et al., 2020). More recently, studies provided insight into the DVT prevalence by using systematic ultrasound screening of the lower limbs. They established a DVT prevalence up to 85.4% in hospitalized COVID-19 patients (Fraissé et al., 2020; Llitjos et al., 2020; Nahum et al., 2020; Ren et al., 2020; Voicu et al., 2020a; Zhang et al., 2020). Timing and number of ultrasound examinations per patient varied according to the study. The highest DVT prevalence was reported by Ren et al. (2020), who performed at least two ultrasound examinations during the intensive care unit (ICU) stay in a Chinese series of 48 patients under prophylactic enoxaparin and showed an overall DVT prevalence of 84.4% but a prevalence of proximal DTV (defined as popliteal or femoral) of 10.4%. In a study of 143 patients admitted to the medical wards, Zhang et al. (2020) showed that 46.1% had DVT, of which 34.8% were proximal. Approximately 50% of the patients received prophylactic or therapeutic anticoagulation before the ultrasound. The median time from admission to detection of DVT was 9 days (Zhang et al., 2020). Patients with DVT had a worse prognosis requiring more often admission to the ICU, 18.2% of the patients versus 3.9% (*p* = 0.005) and having a higher mortality, 34.8% versus 11.7%, *p* = 0.001, compared to patients without DVT. In 56 mechanically ventilated patients, Voicu et al. (2020a) showed a prevalence of 46% of DVT, 50% of them being proximal. Importantly, despite standard unfractionated heparin or enoxaparin prophylaxis, DVT was acquired during ICU stay in 35% of the patients who had a second ultrasound and who were DVT-free on the initial ultrasound (Voicu et al., 2020a). These studies proved that prevalence of DVT was higher that initially suggested, and much higher than in mechanically ventilated influenza patients (i.e., 10.7%) (Obi et al., 2019). However, in non-ICU patients studies performing systematic screening observed lower DVT prevalence of 11.9 with 2.4% being proximal (Santoliquido et al., 2020) and 14.7% among which 1/156 was proximal (Demelo-Rodríguez et al., 2020).

Overall, data from these studies suggested that standard prophylactic anticoagulation was insufficient to prevent DVT and importantly, that ultrasound monitoring should be undertaken to accurately diagnose and treat DVT.

Although routine screening has been performed for DVT using ultrasound, a non-invasive, reliable and cost-effective technique, screening for PE is limited by the difficulties to transport patients in life-threatening conditions to the imaging department and by the adverse effects of computed tomography pulmonary angiography (CTPA) including the high risk of kidney injury and potentially life-threatening allergic reactions. It is not clear how many hospitalized COVID-19 patients present with PE as no study performed routine CTPA. Among retrospective studies reporting PE, a prevalence of 30% was found in 106 patients receiving CTPA for suspicion of PE or other indications, but this high prevalence must be put into perspective with the selection bias inherent to the retrospective nature of the study (Léonard-Lorant et al., 2020). However, one study reporting systematic autopsy studies in patients who died from COVID-19 infection, found that 21% of the patients had PE, and in approximately half of them (8/17), PE was considered the cause of death (Edler et al., 2020).

Several factors can be identified as predisposing to thrombosis in COVID-19 patients. Most patients had underlying conditions including obesity, diabetes, hypertension, ischemic heart disease, associated with hypercoagulable state, endothelial injuries, decreased venous return and immobility, all of which are major factors contributing to thrombosis.

### Hypercoagulability Favoring Thrombosis in COVID-19 Patients

**FIGURE 1:**
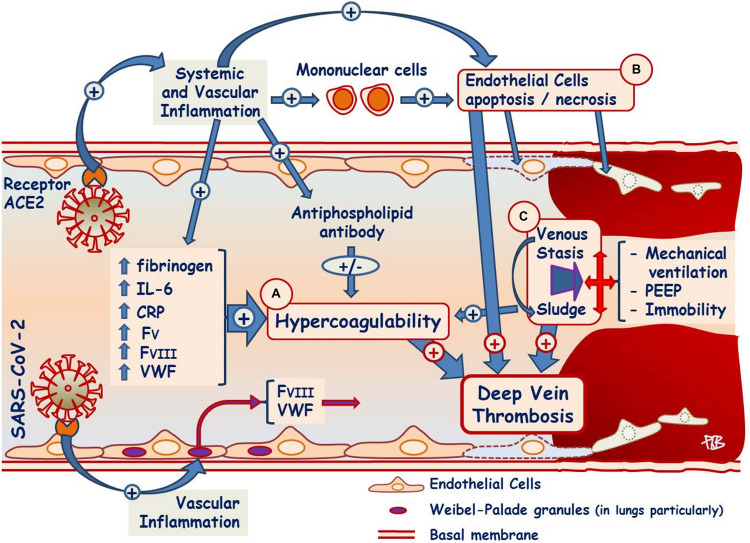
Factors favoring deep vein thrombosis in COVID-19 patients. **(A)** SARS-CoV-2 invades the endothelial cell and produces systemic and vascular inflammation leading to increased fibrinogen, CRP, IL-6, and coagulation factor V, as well as antiphospholipid antibodies possibly through molecular mimicry. Mononuclear cell inflammation, apoptosis, and necrosis were documented in the lungs; in veins, their presence was not specifically studied. SARS-CoV-2 invades the endothelial cell and determines directly and/or through systemic inflammation the exocytosis of von Willebrand factor and factor VIII from Weibel–Palade granules. **(B)** SARS-CoV-2 involves endothelial cells injuries (apoptosis and necrosis). **(C)** Mechanical ventilation, high PEEP, and immobility decrease the blood velocities of the venous return. **(A–C)** Complete the Virchow triad and increase the risk of thrombosis. SARS-CoV-2, severe acute respiratory syndrome–coronavirus 2 virus; IL-6, interleukin-6; CRP, C-reactive-protein; FV, coagulation factor V; FVIII, coagulation factor VIII; VWF, von Willebrand factor; PEEP, positive end-expiratory pressure; ACE2, angiotensin-converting enzyme type 2.

The hypercoagulable state underlies the predisposition to thrombosis as indicated in the first studies in China (Tang et al., 2020), especially by the extremely elevated D-dimers. Tang et al. found a correlation between the D-dimer level and mortality and suggested that anticoagulation may improve survival, as subsequently suggested in a retrospective study including 2773 patients showing increased survival in patients treated with versus without anticoagulation in the United States (Paranjpe et al., 2020). The intense inflammatory reaction supported by the elevated interleukin 6 (IL-6), C-reactive protein, and fibrinogen levels (McElvaney et al., 2020) strongly favors thrombosis. Besides the increase in fibrinogen level, one of the most remarkable laboratory features of the hypercoagulable state in COVID-19 patients is the unusually high von Willebrand factor levels, up to 10 times the upper normal limit and higher than the normal range in 100% of the patients (Panigada et al., 2020; Voicu et al., 2020b); factor VIII level is subsequently very elevated, as factor VIII is transported by von Willebrand factor (Tabatabai et al., 2020; Panigada et al., 2020; Voicu et al., 2020b). Moreover, factor V concentration is also unusually increased (Helms et al., 2020; Voicu et al., 2020b). Both von Willebrand factor and factor VIII are stored in the Weibel–Palade bodies of the endothelial cells, especially in some vascular territories such as lungs, and released during the COVID-19 disease, resulting in high circulating levels (Valentijn et al., 2011). This release may be related to the invasion of the endothelial cell by the virus, which can attach to the angiotensin-converting enzyme type 2, and may lead to endothelial dysfunction or damage and release of the coagulation factors (Panigada et al., 2020; Escher et al., 2020; Figure 1). Other specific pathways such as anticoagulant protein C may also play a role, although the extent of its involvement is still under evaluation. Physiologically, thrombin activates protein C through the formation of a thrombin–thrombomodulin complex on the endothelial surface, enhanced by the endothelial protein C receptor. Therefore, endothelial damage may result in protein C pathway disruption. Its protein C activity was found normal in some studies (Panigada et al., 2020), decreased in others (Tabatabai et al., 2020), or appeared deficient despite normal concentrations of protein C due to the saturation or overwhelming of the pathway by high factor V and VIII concentrations (Voicu et al., 2020b). Similarly, ADAMTS13, the von Willebrand factor–specific protease, seems to be slightly decreased, with a subsequent imbalance of von Willebrand factor/ADAMTS13 axis when considering the strikingly elevated von Willebrand factor levels (Blasi et al., 2020; Delrue et al., 2020; Huisman et al., 2020). Hypoxemia associated with COVID-19–related pneumonia (Sherren et al., 2020) may also enhance hypercoagulability through increased synthesis of hypoxia inducible factor-1α (Marchetti, 2020), which increases procoagulant gene expression (Gupta et al., 2019).

Cardiovascular risk factors predispose to COVID-19 but are also associated with increased predisposition to thrombosis. Obesity is associated with increased tissue factor and P-selectin expression, increased thrombin generation, and platelet activation, whereas antithrombotic pathways such as protein C anticoagulant system, antithrombin, and tissue factor pathway inhibitor are downregulated (Korakas et al., 2020). Diabetes increases the thrombotic risk even further, hyperglycemia increasing the oxidative stress with subsequent thrombin generation and decreasing antithrombin anticoagulant activity, through its non-enzymatic glycation (Ceriello, 2020).

The cytokine storm described in COVID-19 patients is a major pathophysiological bridge between inflammation and thrombosis. Although its complex pathophysiology is beyond the scope of this mini-review, the relationship to thrombosis of one of the main factors involved, IL-6, found in very high levels in COVID-19 patients (Lazzaroni et al., 2020) must be acknowledged. Notably, IL-6 up-regulates fibrinogen levels, activates tissue factor expression, but also increases factor VIII level, through endothelial injury or activation by complex formed by IL-6 and IL6-receptor and subsequent von Willebrand factor release (Joly et al., 2020).

### Endothelial Activation and Lesion Favoring Thrombosis in COVID-19 Patients

Thrombosis may also be favored by endothelial cell activation leading to increased expression of adhesion molecules and production of tissue factor by antiphospholipid antibodies, which are occasionally synthesized in viral infections due to the antigen mimicry of the virus (Ruiz-Irastorza et al., 2010). Lupus anticoagulant was positive in 45% of the 56 COVID-19–infected patients, whereas anticardiolipin and/or β2 glycoprotein-I antibodies were found in 10% of the patients (Harzallah et al., 2020). However, their role in the pathogenesis of COVID-19–related thrombosis remains to be determined, since in a study including 74 mechanically ventilated ICU COVID-19 patients receiving systematic ultrasound DVT screening and CTPA if PE was suspected, 12% had positive anticardiolipin and/or β2 glycoprotein-I antibodies, without difference in prevalence between the 28 patients with a thrombotic event and the 46 patients without a thrombotic event, 5 (18%) versus 4 (9%) respectively, *p* = 0.30 (Siguret et al., 2020).

Neutrophils seem to participate actively in the immunothrombotic process by the increased expression of neutrophil extracellular traps (NETs, extracellular traps of chromatin and microbicidal proteins (Zuo et al., 2020) that activate tissue factor expression by endothelial cells in COVID-19 infection) (Skendros et al., 2020). Moreover, complement fraction C5a increases NET expression and tissue factor expression by neutrophils, participating in the thrombotic process (Skendros et al., 2020). Complement is another potential enhancer of thrombosis, the membrane attack complex C5b-9 being increased in COVID-19–infected patients, in parallel with other endothelial activation markers such as von Willebrand factor, tissue plasminogen activator, and plasminogen activator inhibitor-1 (Cugno et al., 2020).

Finally, the endothelium, which also has antithrombotic functions, may be severely damaged (Magalhaes et al., 2020) due to the underlying vascular mononuclear inflammation, as well as apoptosis of endothelial cells documented by pathology studies especially in the lungs or small bowel vessels (Escher et al., 2020; Varga et al., 2020). These features have not been so far described in veins, but may represent another putative thrombosis-favoring mechanism.

Increased thrombosis may lead to increased consumption of platelets and the onset of thrombocytopenia in 5–41.7% of the patients, percentage varying according to the severity of the disease, being more frequent in patients with more severe disease (58–95%) (Wool and Miller, 2020). More severe thrombocytopenia is encountered in cases of disseminated intravascular coagulation (DIC), diagnosed in 2% of the patients in a study including 400 patients, of which 144 were critically ill (Al-Samkari et al., 2020). Coagulation abnormalities, apart from rare cases of DIC, include mildly prolonged prothrombin time (PT) and thrombin time (TT), and increased fibrin degradation products (FDPs)/D-dimers. Noteworthy, PT prolongation associated with normal or elevated coagulation factor concentrations may be most of the time artifactual, due to unusual hyperfibrinogenemia, depending on PT reagents. Similarly, the interpretation of TT prolongation may be difficult because of the presence of FDP high levels and of heparin.

Moreover, microvascular thrombosis promoting kidney injury and lung microvascular thrombosis promoting hypoxemia (Sherren et al., 2020; Vinayagam and Sattu, 2020) may further increase predisposition to thrombosis.

### Hemorrheologic Factors Favoring Thrombosis

In its most severe form, immobility related to bed rest during hospitalization is present in mechanically ventilated patients requiring muscular paralysis for the treatment of the acute respiratory distress syndrome (ARDS). These patients are immobile and have no muscular contraction and no or very little muscular tone; therefore, venous return is extremely impaired. The half-seated position used in mechanically ventilated patients to improve ventilation parameters introduces another obstacle to venous return, depending on the angle between lower limbs and the body (Zhang et al., 2018), decreasing the blood flow velocity of the venous return and increasing the diameter of the lower limb veins due to venous pooling.

In addition, intubated critically ill patients are ventilated with high levels of positive end-expiratory pressure (PEEP), the median reported PEEP being 12 cmH_2_O (Beloncle et al., 2020) increasing central and peripheral venous pressures. The association of half-seated position and mechanical ventilation increases lower limb venous pressures and decreases the venous return as supported by low maximal velocities in the common femoral vein compared to those in healthy subjects (approximately −45% vs. normal values) (Delis et al., 2004; Needleman et al., 2018). Additionally, increased femoral vein diameters compared to normal (approximately + 31% vs. normal values) consecutive to the increase in hydrostatic pressure in lower limbs veins also contribute to venous stasis and may favor thrombosis. Furthermore, in patients with myocarditis and cardiac failure, low cardiac output may increase venous stasis (Shchedrygina et al., 2020). Moreover, during COVID-19 ARDS, pulmonary microcirculatory thrombosis may occur as a consequence of the disease participating in the increased pulmonary arterial resistance and right ventricular pressures and consequently may decrease velocities of venous return to the heart and contributes to peripheral venous stasis (Ackermann et al., 2020; Edler et al., 2020).

**FIGURE 2:**
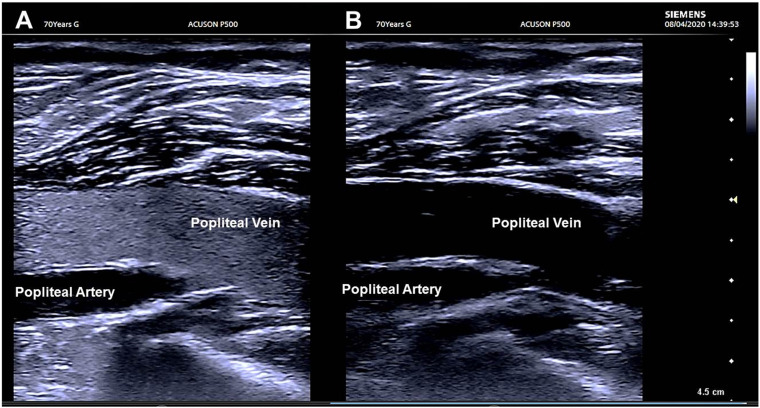
Ultrasound imaging of the popliteal vein. **(A)** The flow is characterized by the presence of a sludge consisting in the increased echogenicity of blood due to the “brightness” of the erythrocyte aggregates occurring in vessels with low blood flow velocities. **(B)** A calf compression is performed that provokes a sudden increase in blood flow velocities in the popliteal vein; blood becomes anechoic with the disappearance of the sludge pattern. The sludge pattern is recovered in less than 20 s after compression. In both panels, the blood is anechoic in the popliteal artery because blood flow velocities are elevated compared to the popliteal vein.

Associated peripheral hemodynamic and hemorrheologic alterations have been observed during duplex ultrasound as pronounced sludge patterns particularly in the lower limbs. These impairments may have resulted from the abnormal “brightness” of erythrocyte aggregates occurring in vessels with lowered blood flow velocities (Stuart and Nash, 1990; Knaggs et al., 2005; Figure 2). The sludge pattern may be considered as a prothrombotic stage by itself (Delis et al., 2004).

### Interpretation of Observations in COVID-19 Patients

Altogether (1) hypercoagulation state of which the most remarkable features are increased fibrinogen, D-dimer, factor VIII, and von Willebrand factor; (2) endothelial lesions due to the viral invasion and vascular inflammation; and (3) immobility, half-seated position, and increased resistance to venous return due to mechanical ventilation PEEP and pulmonary vascular network injuries concur to create the ideal conditions for the occurrence of the Virchow triad (Virchow, 1856; Wolberg et al., 2012) and the consecutive increased DVT prevalence in critically ill COVID-19 patients even under standard prophylactic anticoagulation. COVID-19 plays a major role in thrombus formation in the whole circulatory system by acting on the different actors of the triad of Virchow, Moreover, cytokine storm and organ disorders consecutive to local microvascular thrombosis complete the triad of Virchow to increase the prevalence of DVT. Comorbidities such as diabetes, hypertension, and obesity can increase cytokine storm and hypercoagulability during COVID-19, thus increasing organ dysfunction and predisposition to thrombosis. Therefore, to reduce the thrombotic risk, increased doses of anticoagulants may be required (Marietta et al., 2020; Susen et al., 2020), several regimens being currently evaluated in ongoing randomized trials (Porfidia et al., 2020), whereas some comparative studies showed decreased DVT after increased anticoagulation (Voicu et al., 2020c). Patients presenting low vein blood flow velocities, rising to prethrombotic sludge patterns, do benefit from permanent compression stockings or intermittent leg compression. Anti-inflammatory treatment may also play a role in decreasing endothelial lesions and thus decrease predisposition to thrombosis. Finally, as DVT has a high prevalence, ultrasound screening may be required in order to promptly and accurately diagnose and treat DVT. In case of logistical difficulties and limited availability, ultrasound can be performed according to plasma D-dimer levels as suggested by several studies (Cui et al., 2020; Léonard-Lorant et al., 2020; Voicu et al., 2020b): the threshold of 3,300 ng/mL (Voicu et al., 2020b) similar to 3,000 ng/mL (Cui et al., 2020) and 2,680 ng/mL (Léonard-Lorant et al., 2020) offering a sensitivity of 78% and specificity of 69% for the onset of a thrombotic event defined as DVT or PE.

## Conclusion

In conclusion, hypercoagulable state, endothelial lesions, immobility, and reduced venous blood flows explain the extremely high prevalence of thromboembolic events in mechanically ventilated COVID-19 patients. None of the abnormalities alone in any component of Virchow triad fully explain the high DVT prevalence in mechanically ventilated COVID-19 patients. Initiation of a DVT in lower limbs responds to complex, multifactorial, and interactive processes. Careful monitoring and screening with lower limbs ultrasound based on a high index of suspicion may be necessary to promptly diagnose and treat DVT. Studies are ongoing to establish if higher prophylactic regimens could be more effective in preventing thrombotic events and result in an overall benefit in the outcome of COVID-19 patients.

## Author Contributions

SV and PB wrote the manuscript. CK, AS, BGC, NM, VS, AM, and BM read and amended the final version of the manuscript and provided guidance on the overall direction of the manuscript. All authors critically appraised the final version of the article.

## Conflict of Interest

AM reports personal fees from Orion, Servier, Otsuka, Philips, Sanofi, Adrenomed, Epygon, and Fire 1 and grants and personal fees from 4TEEN4, Abbott, Roche, and Sphingotec. The remaining authors declare that the research was conducted in the absence of any commercial or financial relationships that could be construed as a potential conflict of interest.

## References

[B1] Ackermann M. Verleden S. E. Kuehnel M. Haverich A. Welte T. Laenger F. (2020). Pulmonary vascular endothelialitis, thrombosis, and angiogenesis in Covid-19. *N. Engl. J. Med.* 383 120–128. 10.1056/NEJMoa2015432 32437596 PMC7412750

[B2] Al-Samkari H. Karp Leaf R. S. Dzik W. H. Carlson J. C. T. Fogerty A. E. Waheed A. (2020). COVID-19 and coagulation: bleeding and thrombotic manifestations of SARS-CoV-2 infection. *Blood* 136 489–500. 10.1182/blood.2020006520 32492712 PMC7378457

[B3] Beloncle F. M. Pavlovsky B. Desprez C. Fage N. Olivier P. Y. Asfar P. (2020). Recruitability and effect of PEEP in SARS-Cov-2-associated acute respiratory distress syndrome. *Annals Tntensive Care* 10:55. 10.1186/s13613-020-00675-7 32399901 PMC7215140

[B4] Blasi A. von Meijenfeldt F. A. Adelmeijer J. Calvo A. Ibañez C. Perdomo J. (2020). In vitro hypercoagulability and ongoing in vivo activation of coagulation and fibrinolysis in COVID-19 patients on anticoagulation. *J. Thromb. Haemost.* 18 2646–2653. 10.1111/jth.15043 32762118 PMC7436627

[B5] Ceriello A. (2020). Diabetes, D-dimer and COVID-19: the possible role of glucose control. *Diabetes Metab. Syndrome* 14:1987. 10.1016/j.dsx.2020.10.011 33080539 PMC7557166

[B6] Contou D. Pajot O. Cally R. Logre E. Fraissé M. Mentec H. (2020). Pulmonary embolism or thrombosis in ARDS COVID-19 patients: a french monocenter retrospective study. *PloS One* 15:e0238413 10.1371/journal.pone.0238413 PMC7451560 32853267

[B7] Cui S. Chen S. Li X. Liu S. Wang F. (2020). Prevalence of venous thromboembolism in patients with severe novel coronavirus pneumonia. *J. Thromb. Haemost.* 18 1421–1424. 10.1111/jth.14830 32271988 PMC7262324

[B8] Cugno M. Meroni P. L. Gualtierotti R. Griffini S. Grovetti E. Torri A. (2020). Complement activation and endothelial perturbation parallel COVID-19 severity and activity. *J. Autoimmun.* 29:102560. 10.1016/j.jaut.2020.102560 [Epub ahead of print]. 33139116 PMC7598768

[B9] Delis K. T. Knaggs A. L. Sonecha T. N. Zervas V. Jenkins M. P. Wolfe J. H. (2004). Lower limb venous haemodynamic impairment on dependency: quantification and implications for the “economy class” position. *Thromb. Haemost.* 91 941–950. 10.1160/TH03-12-0754 15116255

[B10] Delrue M. Siguret V. Neuwirth M. Joly B. Beranger N. Sène D. (2020). Von willebrand factor / ADAMTS13 axis and venous thromboembolism in moderate-to-severe COVID-19 patients. *Br. J. Haematol*. 2020:17216 10.1111/bjh.17216 33368196

[B11] Demelo-Rodríguez P. Cervilla-Muñoz E. Ordieres-Ortega L. Parra-Virto A. Toledano-Macías M. Toledo-Samaniego N. (2020). Incidence of asymptomatic deep vein thrombosis in patients with COVID-19 pneumonia and elevated D-dimer levels. *Thromb. Res.* 192 23–26. 10.1016/j.thromres.2020.05.018 32405101 PMC7219400

[B12] Edler C. Schröder A. S. Aepfelbacher M. Fitzek A. Heinemann A. Heinrich F. (2020). Dying with SARS-CoV-2 infection-an autopsy study of the first consecutive 80 cases in hamburg, germany. *Int. J. Legal Med.* 134 1275–1284. 10.1007/s00414-020-02317-w 32500199 PMC7271136

[B13] Escher R. Breakey N. Lämmle B. (2020). Severe COVID-19 infection associated with endothelial activation. *Thromb. Res.* 190:62. 10.1016/j.thromres.2020.04.014 32305740 PMC7156948

[B14] Fraissé M. Logre E. Pajot O. Mentec H. Plantefève G. Contou D. (2020). Thrombotic and hemorrhagic events in critically ill COVID-19 patients: a french monocenter retrospective study. *Crit. Care* 24:275 10.1186/s13054-020-03025-y PMC7265664 32487122

[B15] Goshua G. Pine A. B. Meizlish M. L. Chang C. H. Zhang H. Bahel P. (2020). Endotheliopathy in COVID-19-associated coagulopathy: evidence from a single-centre, cross-sectional study. Lancet. *Haemat.* 7 e575–e582. 10.1016/S2352-3026(20)30216-7 PMC7326446 32619411

[B16] Gupta N. Zhao Y.-Y. Evans C. E. (2019). The stimulation of thrombosis by hypoxia. *Thromb. Res.* 181 77–83. 10.1016/j.thromres.2019.07.013 31376606

[B17] Harzallah I. Debliquis A. Drénou B. (2020). Lupus anticoagulant is frequent in patients with Covid-19: response to reply. *J. Thromb. Haemost.* 2020:32608109. 10.1111/jth.14980 32608109 PMC7361771

[B18] Helms J. Tacquard C. Severac F. Leonard-Lorant I. Ohana M. Delabranche X. (2020). High risk of thrombosis in patients with severe SARS-CoV-2 infection: a multicenter prospective cohort study. *Intensive Care Med.* 46 1089–1098. 10.1007/s00134-020-06062-x 32367170 PMC7197634

[B19] Huisman A. Beun R. Sikma M. Westerink J. Kusadasi N. (2020). Involvement of ADAMTS13 and von willebrand factor in thromboembolic events in patients infected with SARS-CoV-2. *Int. J. Lab. Hematol.* 42 e211–e212. 10.1111/ijlh.13244 32441844 PMC7280565

[B20] Iba T. Levy J. H. Connors J. M. Warkentin T. E. Thachil J. Levi M. (2020). The unique characteristics of COVID-19 coagulopathy. *Crit. Care* 24:360. 10.1186/s13054-020-03077-0 32552865 PMC7301352

[B21] Joly B. S. Siguret V. Veyradier A. (2020). Understanding pathophysiology of hemostasis disorders in critically ill patients with COVID-19. *Intensive Care Med.* 46 1603–1606. 10.1007/s00134-020-06088-1 32415314 PMC7225398

[B22] Klok F. A. Kruip M. van der Meer N. Arbous M. S. Gommers D. Kant K. M. (2020). Incidence of thrombotic complications in critically ill ICU patients with COVID-19. *Thromb. Res.* 191 145–147. 10.1016/j.thromres.2020.04.013 32291094 PMC7146714

[B23] Knaggs A. L. Delis K. T. Mason P. Macleod K. (2005). Perioperative lower limb venous haemodynamics in patients under general anaesthesia. *Br. J. Anaesth.* 94 292–295. 10.1093/bja/aei049 15591327

[B24] Konstantinides S. V. Meyer G. Becattini C. Bueno H. Geersing G. J. Harjola V. P. (2020). ESC scientific document group. 2019 ESC guidelines for the diagnosis and management of acute pulmonary embolism developed in collaboration with the european respiratory society (ERS). *Eur. Heart J.* 41 543–603. 31504429 10.1093/eurheartj/ehz405

[B25] Korakas E. Ikonomidis I. Kousathana F. Balampanis K. Kountouri A. Raptis A. (2020). Obesity and COVID-19: immune and metabolic derangement as a possible link to adverse clinical outcomes. *Am. J. Physiol. Endocrinol. Metab.* 319 E105–E109. 10.1152/ajpendo.00198.2020 32459524 PMC7322508

[B26] Lazzaroni M. G. Piantoni S. Masneri S. Garrafa E. Martini G. Tincani A. (2020). Coagulation dysfunction in COVID-19: the interplay between inflammation, viral infection and the coagulation system. *Blood Rev.* 2020:100745. 10.1016/j.blre.2020.100745 32868115 PMC7444609

[B27] Léonard-Lorant I. Delabranche X. Séverac F. Helms J. Pauzet C. Collange O. (2020). Acute pulmonary embolism in patients with COVID-19 at CT angiography and relationship to d-dimer levels. *Radiology* 296 E189–E191. 10.1148/radiol.2020201561 32324102 PMC7233397

[B28] Llitjos J. F. Leclerc M. Chochois C. Monsallier J. M. Ramakers M. Auvray M. (2020). High incidence of venous thromboembolic events in anticoagulated severe COVID-19 patients. *J. Thromb. Haemost.* 18 1743–1746. 10.1111/jth.14869 32320517 PMC7264774

[B29] Magalhaes G. S. Rodrigues-Machado M. da G. Motta-Santos D. Campagnole-Santos M. J. Santos R. A. S. (2020). Activation of Ang-(1-7)/mas receptor is a possible strategy to treat coronavirus (SARS-CoV-2) infection. *Front. Physiol.* 11:730. 10.3389/fphys.2020.00730 32636762 PMC7318839

[B30] Marchetti M. (2020). COVID-19-driven endothelial damage: complement, HIF-1, and ABL2 are potential pathways of damage and targets for cure. *Ann. Hematol.* 99 1701–1707. 10.1007/s00277-020-04138-8 32583086 PMC7312112

[B31] Marietta M. Ageno W. Artoni A. De Candia E. Gresele P. Marchetti M. (2020). COVID-19 and haemostasis: a position paper from italian society on thrombosis and haemostasis (SISET). *Blood Transfusion = Trasfusione del Sangue* 18 167–169. 10.2450/2020.0083-20 32281926 PMC7250682

[B32] McElvaney O. J. McEvoy N. McElvaney O. F. Carroll T. P. Murphy M. P. Dunlea D. M. (2020). Characterization of the inflammatory response to severe COVID-19 illness. *Am. J. Respir. Crit. Care Med.* 202 812–821. 10.1164/rccm.202005-1583OC 32584597 PMC7491404

[B33] Nahum J. Morichau-Beauchant T. Daviaud F. Echegut P. Fichet J. Maillet J. M. (2020). Venous thrombosis among critically Ill patients with coronavirus disease 2019 (COVID-19). *JAMA Network open* 3:e2010478. 10.1001/jamanetworkopen.2020.10478 32469410 PMC7260620

[B34] Needleman L. Cronan J. J. Lilly M. P. Merli G. J. Adhikari S. Hertzberg B. S. (2018). Ultrasound for lower extremity deep venous thrombosis: multidisciplinary recommendations from the society of radiologists in ultrasound consensus conference. *Circulation* 137 1505–1515. 10.1161/CIRCULATIONAHA.117.030687 29610129

[B35] Obi A. T. Tignanelli C. J. Jacobs B. N. Arya S. Park P. K. Wakefield T. W. (2019). Empirical systemic anticoagulation is associated with decreased venous thromboembolism in critically ill influenza A H1N1 acute respiratory distress syndrome patients. *J. Vasc. Surg. Venous Lymphatic Disord.* 7 317–324. 10.1016/j.jvsv.2018.08.010 30477976

[B36] Panigada M. Bottino N. Tagliabue P. Grasselli G. Novembrino C. Chantarangkul V. (2020). Hypercoagulability of COVID-19 patients in intensive care unit: a report of thromboelastography findings and other parameters of hemostasis. *J. Thromb. Haemost.* 18 1738–1742. 10.1111/jth.14850 32302438 PMC9906150

[B37] Paranjpe I. Fuster V. Lala A. Russak A. J. Glicksberg B. S. Levin M. A. (2020). Association of treatment dose anticoagulation with in-hospital survival among hospitalized patients with COVID-19. *J. Am. Coll. Cardiol.* 76 122–124. 10.1016/j.jacc.2020.05.001 32387623 PMC7202841

[B38] Poissy J. Goutay J. Caplan M. Parmentier E. Duburcq T. Lassalle F. (2020). Pulmonary embolism in patients with COVID-19: awareness of an increased prevalence. *Circulation* 142 184–186. 10.1161/CIRCULATIONAHA.120.047430 32330083

[B39] Porfidia A. Valeriani E. Pola R. Porreca E. Rutjes A. Di Nisio M. (2020). Venous thromboembolism in patients with COVID-19: systematic review and meta-analysis. *Thromb. Res.* 196 67–74. 10.1016/j.thromres.2020.08.020 32853978 PMC7420982

[B40] Ren B. Yan F. Deng Z. Zhang S. Xiao L. Wu M. (2020). Extremely high incidence of lower extremity deep venous thrombosis in 48 patients with severe COVID-19 in wuhan. *Circulation* 142 181–183. 10.1161/CIRCULATIONAHA.120.047407 32412320

[B41] Ruiz-Irastorza G. Crowther M. Branch W. Khamashta M. A. (2010). Antiphospholipid syndrome. *Lancet* 376 1498–1509. 10.1016/S0140-6736(10)60709-X 20822807

[B42] Santoliquido A. Porfidia A. Nesci A. De Matteis G. Marrone G. Porceddu E. (2020). Incidence of deep vein thrombosis among non-ICU patients hospitalized for COVID-19 despite pharmacological thromboprophylaxis. *J. Thromb. Haemost.* 18 2358–2363. 10.1111/jth.14992 32633068 PMC7361278

[B43] Shchedrygina A. Nagel E. Puntmann V. O. Valbuena-Lopez S. (2020). COVID-19 myocarditis and prospective heart failure burden. *Exp. Rev Cardiovasc Ther.* 2020 1–10. 10.1080/14779072.2021.1844005 33119418

[B44] Sherren P. B. Ostermann M. Agarwal S. Meadows C. Ioannou N. Camporota L. (2020). COVID-19-related organ dysfunction and management strategies on the intensive care unit: a narrative review. *Br. J. Anaesth.* 125 912–925. 10.1016/j.bja.2020.08.050 32988604 PMC7833857

[B45] Siguret V. Voicu S. Neuwirth M. Delrue M. Gayat E. Stépanian A. (2020). Are antiphospholipid antibodies associated with thrombotic complications in critically ill COVID-19 patients? *Thromb. Res.* 195 74–76. 10.1016/j.thromres.2020.07.016 32663703 PMC7342042

[B46] Skendros P. Mitsios A. Chrysanthopoulou A. Mastellos D. C. Metallidis S. Rafailidis P. (2020). Complement and tissue factor-enriched neutrophil extracellular traps are key drivers in COVID-19 immunothrombosis. *J. Clin. Investigat.* 130 6151–6157. 10.1172/JCI141374 32759504 PMC7598040

[B47] Stuart J. Nash G. B. (1990). Technological advances in blood rheology. *Crit. Rev. Clin. Lab. Sci.* 28 61–93. 10.3109/10408369009105898 2121160

[B48] Susen S. Tacquard C. A. Godon A. Mansour A. Garrigue D. Nguyen P. (2020). Prevention of thrombotic risk in hospitalized patients with COVID-19 and hemostasis monitoring. *Crit. Care* 24:364. 10.1186/s13054-020-03000-7 32560658 PMC7303590

[B49] Tabatabai A. Rabin J. Menaker J. Madathil R. Galvagno S. Menne A. (2020). Factor VIII and functional protein C activity in critically Ill patients with coronavirus disease 2019: a case series. *A A pract.* 14:e01236. 10.1213/XAA.0000000000001236 32539272 PMC7242090

[B50] Tang N. Bai H. Chen X. Gong J. Li D. Sun Z. (2020). Anticoagulant treatment is associated with decreased mortality in severe coronavirus disease 2019 patients with coagulopathy. *J. Thromb. Haemost.* 18 1094–1099. 10.1111/jth.14817 32220112 PMC9906401

[B51] Tavazzi G. Civardi L. Caneva L. Mongodi S. Mojoli F. (2020). Thrombotic events in SARS-CoV-2 patients: an urgent call for ultrasound screening. *Intensive Care Med.* 46 1121–1123. 10.1007/s00134-020-06040-3 32322918 PMC7175449

[B52] Valentijn K. M. Sadler J. E. Valentijn J. A. Voorberg J. Eikenboom J. (2011). Functional architecture of weibel-palade bodies. *Blood* 117 5033–5043. 10.1182/blood-2010-09-267492 21266719 PMC3109530

[B53] Varga Z. Flammer A. J. Steiger P. Haberecker M. Andermatt R. Zinkernagel A. S. (2020). Endothelial cell infection and endotheliitis in COVID-19. *Lancet* 395 1417–1418. 10.1016/S0140-6736(20)30937-5 32325026 PMC7172722

[B54] Vinayagam S. Sattu K. (2020). SARS-CoV-2 and coagulation disorders in different organs. *Life Sci.* 260:118431. 10.1016/j.lfs.2020.118431 32946915 PMC7490584

[B55] Virchow R. (1856). *Gesammalte abhandlungen zur wissenschaftlichen medtzin.* Frankfurt: Medinger Sohn & Co, 219–732.

[B56] Voicu S. Bonnin P. Stépanian A. Chousterman B. G. Le Gall A. Malissin I. (2020a). High prevalence of deep vein thrombosis in mechanically ventilated COVID-19 patients. *J. Am. Coll. Cardiol.* 76 480–482. 10.1016/j.jacc.2020.05.053 32479784 PMC7260513

[B57] Voicu S. Delrue M. Chousterman B. G. Stépanian A. Bonnin P. Malissin I. (2020b). Imbalance between procoagulant factors and natural coagulation inhibitors contributes to hypercoagulability in the critically ill COVID-19 patient: clinical implications. *Eur. Rev. Med. Pharmacol. Sci.* 24 9161–9168. 10.26355/eurrev_202009_22866 32965009

[B58] Voicu S. Chousterman B. G. Bonnin P. Deye N. Malissin I. Le Gall A. (2020c). Increased anticoagulation reduces proximal deep vein thrombosis in mechanically ventilated COVID-19 patients. *J. Infect.* [Epub ahead of print]. 10.1016/j.jacc.2020.05.053 PMC7260513 32479784

[B59] Wolberg A. S. Aleman M. M. Leiderman K. Machlus K. R. (2012). Procoagulant activity in hemostasis and thrombosis: virchow’s triad revisited. *Anesth. Analgesia* 114 275–285. 10.1213/ANE.0b013e31823a088c 22104070 PMC3264782

[B60] Wool G. D. Miller J. L. (2020). The impact of COVID-19 disease on platelets and coagulation. *Pathobiology* 13 1–13. 10.1159/000512007 [Epub ahead of print]. 33049751 PMC7649697

[B61] Zhang L. Feng X. Zhang D. Jiang C. Mei H. Wang J. (2020). Deep vein thrombosis in hospitalized patients with COVID-19 in wuhan. China Prevalence, Risk Factors, and Outcome. *Circulation* 142 114–128. 10.1161/CIRCULATIONAHA.120.046702 32421381

[B62] Zhang Q. Wang X. Su L. Zhang H. Chai W. Chao Y. (2018). Relationship between inferior vena cava diameter ratio and central venous pressure. *J. Clin. Ultrasound* 46 450–454. 10.1002/jcu.22586 29527693

[B63] Zuo Y. Zuo M. Yalavarthi S. Gockman K. Madison J. A. Shi H. (2020). Neutrophil extracellular traps and thrombosis in COVID-19. *J. Thromb. Thrombolysis* 2020 1–8. 10.1007/s11239-020-02324-z 33151461 PMC7642240

